# Supplementary material for: Immune Checkpoint Inhibitor-Induced Pancreatic Injury (ICI-PI) in Adult Cancer Patients: A Systematic Review and Meta-Analysis
Source: Cancers (Basel). 2025 Mar 24;17(7):1080. doi: 10.3390/cancers17071080 (PMC11987741; doi:10.3390/cancers17071080)
Supplement: Supplementary file 1 [file cancers-17-01080-s001.zip › cancers-3462786-supplementary.pdf]

SUPPLEMENTARY MATERIALS

Table S1: PRISMA 2020 Checklist 1

Table S2: PRISMA 2020 for Abstract Checklist 5

Table S3: Full Search Strategy 6

Table S4: Screening parameters, according to the prevalence checklist of related JBI critical appraisal tool and the resulting score for risk of bias of each. 7

Table S5: Reasons for exclusion after full-text review 9

Figure S1: Concurrent immune-related adverse events (irAEs) involving other organs in patients diagnosed with immune-related pancreatitis (ir-pancreatitis). 14

Figure S2: Management for long-term complication diabetes mellitus. (A) insulin usage and (B) oral hypoglycemic agents. 15

Figure S3: Frequency of (A) Permanent discontinuation of immune checkpoint inhibiton (ICI), (B) Resumption of ICI therapy, and (C) Recurrence of immune-related pancreatitis (ir-pancreatitis) following ICI therapy resumption 16

Figure S4: Objective response rate of patients with immune-related pancreatitis.. 17

Figure S5: Leave-one-out and funnel plots of immune-related pancreatitis (ir-pancreatitis). (A) Frequency and (B) Chronic complications 18

Article: Immune Checkpoint Inhibitor-Induced Pancreatic Injury (ICI-PI) in Adult Cancer Patients: A Systematic Review and Meta-Analysis

Authors: Cha Len Lee [1,2,*], Israt Jahan Riya [3], Ifrat Jahan Piya [3], Thiago Pimentel Muniz [2], Marcus Otho Butler [2] and Samuel David Saibil [2]

1 Department of Medical Oncology and Hematology, CancerCare Manitoba, University of Manitoba, Winnipeg, MB R2H 2A6, Canada
2 Division of Medical Oncology and Hematology, Princess Margaret Cancer Center, University of Toronto, University Health Network, Toronto, ON M5S 1Z5, Canada; sam.saibil@uhn.ca (S.D.S.)
3 Dhaka Medical College Hospital, Dhaka 1000, Bangladesh
* Correspondence: lfchalen@yahoo.com

Table S1: PRISMA 2020 Checklist.

Section and Topic	Item #	Checklist item	Location where item is reported
TITLE			
Title	1	Identify the report as a systematic review.	Page 1
ABSTRACT			
Abstract	2	See the PRISMA 2020 for Abstracts checklist.	Supplementary Material Table S2
INTRODUCTION			
Rationale	3	Describe the rationale for the review in the context of existing knowledge.	Page 2
Objectives	4	Provide an explicit statement of the objective(s) or question(s) the review addresses.	Page 2
METHODS			
Eligibility criteria	5	Specify the inclusion and exclusion criteria for the review and how studies were grouped for the syntheses.	Page 3
Information sources	6	Specify all databases, registers, websites, organisations, reference lists and other sources searched or consulted to identify studies. Specify the date when each source was last searched or consulted.	Page 3
Search strategy	7	Present the full search strategies for all databases, registers and websites, including any filters and limits used.	Supplementary Material Table S3
Selection process	8	Specify the methods used to decide whether a study met the inclusion criteria of the review, including how many reviewers screened each record and each report retrieved, whether they worked independently, and if applicable, details of automation tools used in the process.	Page 3
Data collection process	9	Specify the methods used to collect data from reports, including how many reviewers collected data from each report, whether they worked independently, any processes for obtaining or confirming data from study investigators, and if applicable, details of automation tools used in the process.	Page 3
Data items	10a	List and define all outcomes for which data were sought. Specify whether all results that were compatible with each outcome domain in each study were sought (e.g. for all measures, time points, analyses), and if not, the methods used to decide which results to collect.	Page 3
	10b	List and define all other variables for which data were sought (e.g. participant and intervention characteristics, funding sources). Describe any assumptions made about any missing or unclear information.	Pages 3-4
Study risk of bias assessment	11	Specify the methods used to assess risk of bias in the included studies, including details of the tool(s) used, how many reviewers assessed each study and whether they worked	Pages 3-4

Section and Topic	Item #	Checklist item	Location where item is reported
		independently, and if applicable, details of automation tools used in the process.	
Effect measures	12	Specify for each outcome the effect measure(s) (e.g. risk ratio, mean difference) used in the synthesis or presentation of results.	Page 3
Synthesis methods	13a	Describe the processes used to decide which studies were eligible for each synthesis (e.g. tabulating the study intervention characteristics and comparing against the planned groups for each synthesis (item #5)).	Page 3
	13b	Describe any methods required to prepare the data for presentation or synthesis, such as handling of missing summary statistics, or data conversions.	Page 3
	13c	Describe any methods used to tabulate or visually display results of individual studies and syntheses.	Pages 3-4
	13d	Describe any methods used to synthesize results and provide a rationale for the choice(s). If meta-analysis was performed, describe the model(s), method(s) to identify the presence and extent of statistical heterogeneity, and software package(s) used.	Pages 3-4
	13e	Describe any methods used to explore possible causes of heterogeneity among study results (e.g. subgroup analysis, meta-regression).	Pages 3-4, 7
	13f	Describe any sensitivity analyses conducted to assess robustness of the synthesized results.	Pages 3-4, 7
Reporting bias assessment	14	Describe any methods used to assess risk of bias due to missing results in a synthesis (arising from reporting biases).	Pages 3-4
Certainty assessment	15	Describe any methods used to assess certainty (or confidence) in the body of evidence for an outcome.	Pages 3-4
RESULTS			
Study selection	16a	Describe the results of the search and selection process, from the number of records identified in the search to the number of studies included in the review, ideally using a flow diagram.	Page 4
	16b	Cite studies that might appear to meet the inclusion criteria, but which were excluded, and explain why they were excluded.	Supplementary Material Table S5.
Study characteristics	17	Cite each included study and present its characteristics.	Pages 4-6
Risk of bias in studies	18	Present assessments of risk of bias for each included study.	Supplementary Material Table S4
Results of individual studies	19	For all outcomes, present, for each study: (a) summary statistics for each group (where appropriate) and (b) an effect estimate and its precision (e.g. confidence/credible interval), ideally	Pages 4-7

Section and Topic	Item #	Checklist item	Location where item is reported
		using structured tables or plots.	
Results of syntheses	20a	For each synthesis, briefly summarise the characteristics and risk of bias among contributing studies.	Page 7
	20b	Present results of all statistical syntheses conducted. If meta-analysis was done, present for each the summary estimate and its precision (e.g. confidence/credible interval) and measures of statistical heterogeneity. If comparing groups, describe the direction of the effect.	Page 4-7
	20c	Present results of all investigations of possible causes of heterogeneity among study results.	Page 4-7
	20d	Present results of all sensitivity analyses conducted to assess the robustness of the synthesized results.	Pages 7
Reporting biases	21	Present assessments of risk of bias due to missing results (arising from reporting biases) for each synthesis assessed.	Page 7
Certainty of evidence	22	Present assessments of certainty (or confidence) in the body of evidence for each outcome assessed.	Pages 7
DISCUSSION			
Discussion	23a	Provide a general interpretation of the results in the context of other evidence.	Page 7
	23b	Discuss any limitations of the evidence included in the review.	Page 9
	23c	Discuss any limitations of the review processes used.	Page 9
	23d	Discuss implications of the results for practice, policy, and future research.	Pages 9-10
OTHER INFORMATION			
Registration and protocol	24a	Provide registration information for the review, including register name and registration number, or state that the review was not registered.	Page 2
	24b	Indicate where the review protocol can be accessed, or state that a protocol was not prepared.	Page 4
	24c	Describe and explain any amendments to information provided at registration or in the protocol.	Not applicable
Support	25	Describe sources of financial or non-financial support for the review, and the role of the funders or sponsors in the review.	Page 10
Competing interests	26	Declare any competing interests of review authors.	Page 10
Availability of data, code and other materials	27	Report which of the following are publicly available and where they can be found: template data collection forms; data extracted from included studies; data used for all analyses; analytic code; any other materials used in the review.	Page 10

Table S2: PRISMA 2020 for Abstract Checklist.

Section and Topic	Item #	Checklist item	Reported (Yes/No)
TITLE			
Title	1	Identify the report as a systematic review.	Yes
BACKGROUND			
Objectives	2	Provide an explicit statement of the main objective(s) or question(s) the review addresses.	Yes
METHODS			
Eligibility criteria	3	Specify the inclusion and exclusion criteria for the review.	No
Information sources	4	Specify the information sources (e.g. databases, registers) used to identify studies and the date when each was last searched.	Yes
Risk of bias	5	Specify the methods used to assess risk of bias in the included studies.	No
Synthesis of results	6	Specify the methods used to present and synthesise results.	Yes
RESULTS			
Included studies	7	Give the total number of included studies and participants and summarise relevant characteristics of studies.	Yes
Synthesis of results	8	Present results for main outcomes, preferably indicating the number of included studies and participants for each. If meta-analysis was done, report the summary estimate and confidence/credible interval. If comparing groups, indicate the direction of the effect (i.e. which group is favoured).	Yes
DISCUSSION			
Limitations of evidence	9	Provide a brief summary of the limitations of the evidence included in the review (e.g. study risk of bias, inconsistency and imprecision).	Yes
Interpretation	10	Provide a general interpretation of the results and important implications.	Yes
OTHER			
Funding	11	Specify the primary source of funding for the review.	Not applicable
Registration	12	Provide the register name and registration number.	Yes

Table S3: Full Search Strategy.

Databases	Search Terms	Identified records
PubMed	("immunotherapy"[MeSH Terms] OR "ICB" OR "ICI" OR "immune-related" OR irAE*) AND (pancreatitis OR pancrea* OR "exocrine pancreatic insufficiency") AND ((steroid*) OR (fluid*) OR management OR discontinuation OR diabetes OR antibiotic* OR outcomes) NOT ((Plants[MESH] OR Animals[MESH) NOT Humans[MESH]) NOT review[pt]	1393
PubMed	Apply filter time limit 2010-2024 (Ipilimumab was the first immune checkpoint inhibitor approved in 2011)	832
EMBASE	('immunotherapy'/exp OR 'icb':ti,ab OR 'ici':ti,ab OR 'immune-related':ti,ab OR irae:ti,ab) AND ('pancreatitis'/exp OR pancreas:ti,ab OR 'exocrine pancreatic insufficiency'/exp) AND (steroid:ti,ab OR fluid:ti,ab OR management:ti,ab OR discontinuation:ti,ab OR diabetes:ti,ab OR antibiotic:ti,ab OR outcomes:ti,ab) AND [english]/lim AND [2010-2024]/py	1040
Cochrane Library	("immunotherapy" OR "ICB" OR "ICI" OR "immune-related" OR irAE) AND (pancreatitis OR pancreas OR "exocrine pancreatic insufficiency") AND (steroid OR fluid OR management OR discontinuation OR diabetes OR antibiotic OR outcomes)	239
	Total records:	2111

Table S4: Screening parameters, according to the prevalence checklist of related JBI critical appraisal tool and the resulting score for risk of bias of each. Questions codes (Q): Q1-Were the two groups similar and recruited from the same population? Q2-Were the exposures measured similarly to assign people to both exposed and unexposed groups? Q3-Was the exposure measured in a valid and reliable way? Q4-Were confounding factors identified? Q5-Were strategies to deal with confounding factors stated? Q6-Were the groups/participants free of the outcome at the start of the study (or at the moment of exposure)? Q7-Were the outcomes measured in a valid and reliable way? Q8-Was the follow up time reported and sufficient to be long enough for outcomes to occur? Q9-Was follow up complete, and if not, were the reasons to loss to follow up described and explored? Q10-Were strategies to address incomplete follow up utilized? Q11-Was appropriate statistical analysis used? Y-Yes; N-No; U-unclear; NA-Not applicable.

Study	Q1	Q2	Q3	Q4	Q5	Q6	Q7	Q8	Q9	Q10	Q11	Overall appraisal
Abu-Sbeih, 2019[1]	Y	U	Y	Y	Y	Y	Y	Y	Y	NA	Y	9/11
Akazawa, 2014[2]	Y	U	Y	Y	Y	Y	Y	Y	Y	N*A*	Y	9/11
Byun, 2020[3]	N	NA	Y	Y	Y	Y	Y	Y	Y	NA	Y	8/11
Eshet, 2018[4]	Y	U	Y	Y	Y	Y	Y	Y	Y	NA	Y	9/11
Eyada, 2021[5]	Y	U	Y	Y	Y	Y	Y	Y	Y	NA	U	8/11
Gleeson, 2023[6]	Y	U	Y	Y	Y	Y	Y	Y	Y	NA	U	8/11
Grimmelmann, 2021[7]	N	NA	Y	Y	Y	Y	Y	Y	Y	NA	Y	8/11
Hori, 2024[8]	Y	U	Y	Y	Y	Y	Y	Y	Y	NA	Y	9/11
Jeun, 2023[9]	N	NA	Y	Y	Y	Y	Y	Y	Y	NA	Y	8/11
Matsukane, 2023[10]	N	NA	Y	Y	Y	Y	Y	Y	Y	NA	Y	8/11
Nagao, 2024[11]	Y	U	Y	Y	Y	Y	Y	Y	Y	NA	Y	9/11
Nagpal, 2020[12]	Y	U	Y	Y	Y	Y	Y	Y	Y	NA	U	8/11
Ngamphaiboon, 2021[13]	Y	U	Y	Y	Y	Y	Y	Y	Y	NA	Y	9/11
Nizam, 2024[14]	Y	U	Y	Y	Y	Y	Y	Y	Y	NA	Y	9/11
Owen, 2021[15]	Y	U	Y	Y	Y	Y	Y	Y	Y	NA	Y	9/11
Pollack, 2018[16]	Y	Y	Y	Y	Y	Y	Y	Y	Y	NA	Y	10/11
Ruf, 2024[17]	Y	U	Y	Y	Y	Y	Y	Y	Y	NA	Y	9/11
Satish, 2023[18]	Y	U	Y	Y	Y	U	Y	Y	Y	NA	Y	8/11
Shah, 2019[19]	Y	U	Y	Y	Y	U	Y	Y	Y	NA	Y	8/11
Shi, 2022[20]	Y	U	Y	Y	Y	Y	Y	Y	Y	NA	Y	9/11
Shirwaikar, 2021[21]	Y	U	Y	Y	Y	Y	Y	Y	Y	NA	Y	9/11

Townsend, 2023[22]	Y	U	Y	Y	Y	Y	Y	Y	Y	NA	Y	9/11
Tsang, 2019[23]	Y	U	Y	Y	Y	Y	Y	Y	Y	NA	Y	9/11
Wei, 2024[24]	Y	U	Y	Y	Y	Y	Y	Y	Y	NA	Y	9/11
Zhang, 2023[25]	Y	U	Y	Y	Y	Y	Y	Y	Y	NA	Y	9/11

Table S5: Reasons for exclusion after full-text review.

No.	Year	Author	Title	Reasons of Exclusion
1	2019	Abu-Sbeih, H.	Clinical Characteristics and Outcomes of Immune Checkpoint Inhibitor-induced Pancreatic Injury	Only abstract available
2	2018	Abu-Sbeih, H	Clinical characteristics and outcomes of immune checkpoint inhibitor-induced pancreatic injury	Only abstract available
3	2022	Satish, D.	Exocrine and Endocrine Pancreatic Insufficiency Induced by Immune Checkpoint Inhibitors: A Case-Control Study	Only abstract available
4	2024	Hori, Y.	Incidence of Pancreatic Injury and Pancreatitis in Patients Treated with Immune Checkpoint Inhibitors	Only abstract available
5	2021	Townsend, M.J.	Pancreatic insufficiency and time to disease progression following immune checkpoint inhibitor pancreatic injury in advanced cancers	Only abstract available
6	2022	Y, Zhang	Pancreatic Adverse Events Associated With Immune Checkpoint Inhibitors: A Large-Scale Pharmacovigilance Analysis.	Registry study
7	2022	Abreo, M.	Immune Checkpoint Inhibitor-induced (Type 3) Autoimmune Pancreatitis: Clinical Profile and Natural History Based On Serial Pancreas Volumetry	Missing management details
8	2018	Simonaggio	Safety assessment of anti-PD(L)1 rechallenge after immune-related adverse events	Missing management details
9	2024	Itani, J.	Clinical feature of immune checkpoint inhibitor induced pancreatic injury	Missing management details
10	2024	L, Wu	Checkpoint Inhibitor-Associated Autoimmune Diabetes Mellitus Is Characterized by C-peptide Loss and Pancreatic Atrophy.	No outcome of interest

11	2021	AL, Zhang	Coexistence of Immune Checkpoint Inhibitor-Induced Autoimmune Diabetes and Pancreatitis.	No outcome of interest
12	2018	ML, Gauci	Occurrence of type 1 and type 2 diabetes in patients treated with immunotherapy (anti-PD-1 and/or anti-CTLA-4) for metastatic melanoma: a retrospective study.	No outcome of interest
13	2021	Jeun, R.	Pancreatic volumes in immune checkpoint inhibitor-induced diabetes	No outcome of interest
14	2020	Asher, N.	Real world outcomes of ipilimumab and nivolumab in patients with metastatic melanoma	No outcome of interest
15	2020	Shah, P.	Response to immune checkpoint inhibitor (ICI) rechallenge after high-grade immune related adverse events (irAE) in patients (pts) with metastatic melanoma (MM)	No outcome of interest
16	2022	EA, Basak	The course of C-peptide levels in patients developing diabetes during anti-PD-1 therapy.	No outcome of interest
17	2023	Thomas, A.S.	Type 3 Autoimmune Pancreatitis: Clinical Profile and Response to Steroids	No outcome of interest
18	2020	Chandra, G.S.	58P Real-world data of incidence of immune related toxicities with single agent immuno oncology drugs at a single center	No outcome of interest
19	2019	Castillo, R.	311 Retrospective Analysis of Patients Receiving Immune Checkpoint Inhibitors Presenting to the Emergency Department	No outcome of interest
20	2019	León, P.	Adverse autoimmune events in cancer patients treated with immunotherapy. Analysis of cases between 2011 and 2018	No outcome of interest
21	2022	Liang, K.-L.	An Oncology Urgent Care Clinic for the Management of Immune-Related Adverse Events: A Descriptive Analysis	No outcome of interest
22	2016	L, Hofmann; A	Cutaneous, gastrointestinal, hepatic, endocrine, and renal side-effects of anti-PD-1 therapy.	No outcome of interest

23	2021	Dimitriou, F.	Frequency, treatment and outcome of immune-related toxicities in patients with immune-checkpoint inhibitors for advanced melanoma: Results from an institutional database analysis	No outcome of interest
24	2020	Siddiqui, B.	Immune checkpoint therapy (ICT) re-challenge after immune-related adverse events (irAEs) in genitourinary cancers (GUC)	No outcome of interest
25	2017	Cabanillas, G.	Immune related adverse events and their treatment in melanoma patients receiving ipilimumab	No outcome of interest
26	2018	Romero, R.	Immune related adverse events from immune checkpoint inhibitors: A retrospective analysis from 2004-2017 at the university of north carolina at chapel hill	No outcome of interest
27	2023	Niu, C.	Immune-related Adverse Effects of Immune Checkpoint Inhibitors for Colorectal Cancer: A Multicenter Cohort Study	No outcome of interest
28	2022	Prather, L.L.	Immune-related adverse events and immunotherapy efficacy in patients with cancer: A retrospective study	No outcome of interest
29	2018	Balaji, A.	Immune-related adverse events requiring inpatient management: Spectrum of toxicity, treatment, and outcomes	No outcome of interest
30	2018	Shoushtari, AN	Measuring toxic effects and time to treatment failure for nivolumab plus ipilimumab in melanoma	No outcome of interest
31	2022	Owaki, S.	MO37-1 Treatment outcome and relapse of serious immune-related adverse events in patients with solid malignancies	No outcome of interest
32	2019	Zhao, D.	Molecular and clinical features of hospital admissions and immunotherapy related adverse events of immune checkpoint inhibitors in thoracic malignancies	No outcome of interest
33	2023	Mashni, O.K.	Retrospective study evaluating immune-related adverse events in cancer patients treated with pembrolizumab	No outcome of interest

34	2019	CR, Stroud	Tocilizumab for the management of immune mediated adverse events secondary to PD-1 blockade.	No outcome of interest
35	2022	Pathmanathan, S.	Toxicity and response to ipilimumab and nivolumab in elderly patients with metastatic melanoma: A multicenter retrospective analysis.	No outcome of interest
36	2022	Pathmanathan, S.	Toxicity and response to ipilimumab and nivolumab in older patients with metastatic melanoma: A multicentre retrospective analysis	No outcome of interest
37	2018	E, Hinchcliff	Characteristics and outcomes of patients with recurrent ovarian cancer undergoing early phase immune checkpoint inhibitor clinical trials.	No outcome of interest
38	2022	Watson, A.S.	Association of Immune-Related Adverse Events, Hospitalization, and Therapy Resumption with Survival among Patients with Metastatic Melanoma Receiving Single-Agent or Combination Immunotherapy	No outcome of interest
39	2018	Bai, X.	Better efficacy of PD-1 antibody predicted by immune-related adverse effects is impaired by high dose steroids	No outcome of interest
40	2021	Patrinely, J.R.	Chronic immune-related adverse events following adjuvant anti-PD-1 therapy for high-risk resected melanoma	No outcome of interest
41	2018	Eshet, Y.	Anti PD-1 therapy induced pancreatic atrophy and its clinical significance	No outcomes of interest
42	2022	Tong, J.	Long-Term Toxicities of Immune Checkpoint Inhibitor (ICI) in Melanoma Patients	No patient with pancreatitis
43	2019	F, Alessandrino	Frequency and imaging features of abdominal immune-related adverse events in metastatic lung cancer patients treated with PD-1 inhibitor.	No patient with pancreatitis
44	2018	NM, Villa	Endocrinopathies with use of cancer immunotherapies.	No patient with pancreatitis

45	2021	Sengul Samanci, N.	Immune-related adverse events associated with immune checkpoint inhibitors in patients with cancer	No patient with pancreatitis
46	2021	Nice, L.	Assessment of hospitalization rates for immune-related adverse events with immune checkpoint inhibitors	No patient with pancreatitis
47	2023	Brongiel, S.	Management of Steroid-Refractory Gastrointestinal Immune-Related Adverse Events	No patient with pancreatitis
48	2023	Tomsitz, D.	Steroid-Refractory Immune-Related Adverse Events Induced by Checkpoint Inhibitors	Overlapping population
49	2021	Townsend, M.J.	Etiology And Outcomes of Pancreatic Enzymes Elevations with the Use of Immune Checkpoint Inhibitors in Advanced Cancers	Overlapping population
50	2017	Pollack, M.	Safety of resuming anti-PD-1 (aPD1) in patients (pts) with immune-related adverse events (irAEs) during combined anti-CTLA-4 (aCTLA4) and aPD1 in metastatic melanoma (MM)	Overlapping population
51	2022	Thomas, A.S.	Autoimmune Pancreatitis Secondary to Immune Checkpoint Inhibitor Therapy (Type 3 AIP): Insights into a New Disease from Clinical Review and Serial Pancreatic Imaging	Update publication is available
52	2018	Fujii, T.	Incidence of immune-related adverse events and its association with treatment outcomes: the MD Anderson Cancer Center experience	Wrong population

Study	Total	Events	Proportion	95% C.I.	Weight
Abu−Sbeih 2019	82	75	91.46	[83.20; 96.50]	13.7%
Eshet 2018	31	19	61.29	[42.19; 78.15]	13.0%
Eyada 2021	85	31	36.47	[26.29; 47.62]	13.8%
Nagao 2024	19	18	94.74	[73.97; 99.87]	12.3%
Satish 2023	23	21	91.30	[71.96; 98.93]	12.6%
Tsang 2019	10	10	100.00	[69.15; 100.00]	11.0%
Wei 2024	10	5	50.00	[18.71; 81.29]	11.0%
Zhang 2023	25	18	72.00	[50.61; 87.93]	12.7%
**Random effects model**	**285**	**197**	**78.85**	**[59.32; 93.18]**	**100.0%**

Heterogeneity: $I^2 = 93\%$, $\tau^2 = 0.0835$, $\chi^2_7 = 97.87$ ($p < 0.01$)

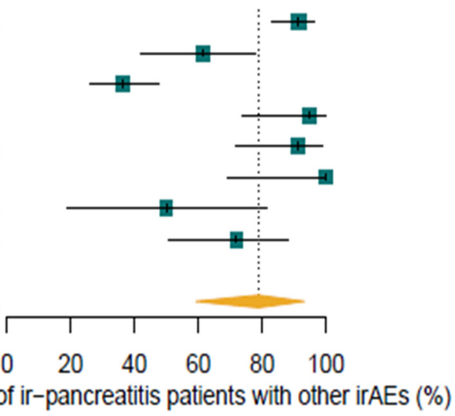

Figure S1: Concurrent immune-related adverse events (irAEs) involving other organs in patients diagnosed with immune-related pancreatitis (ir-pancreatitis).

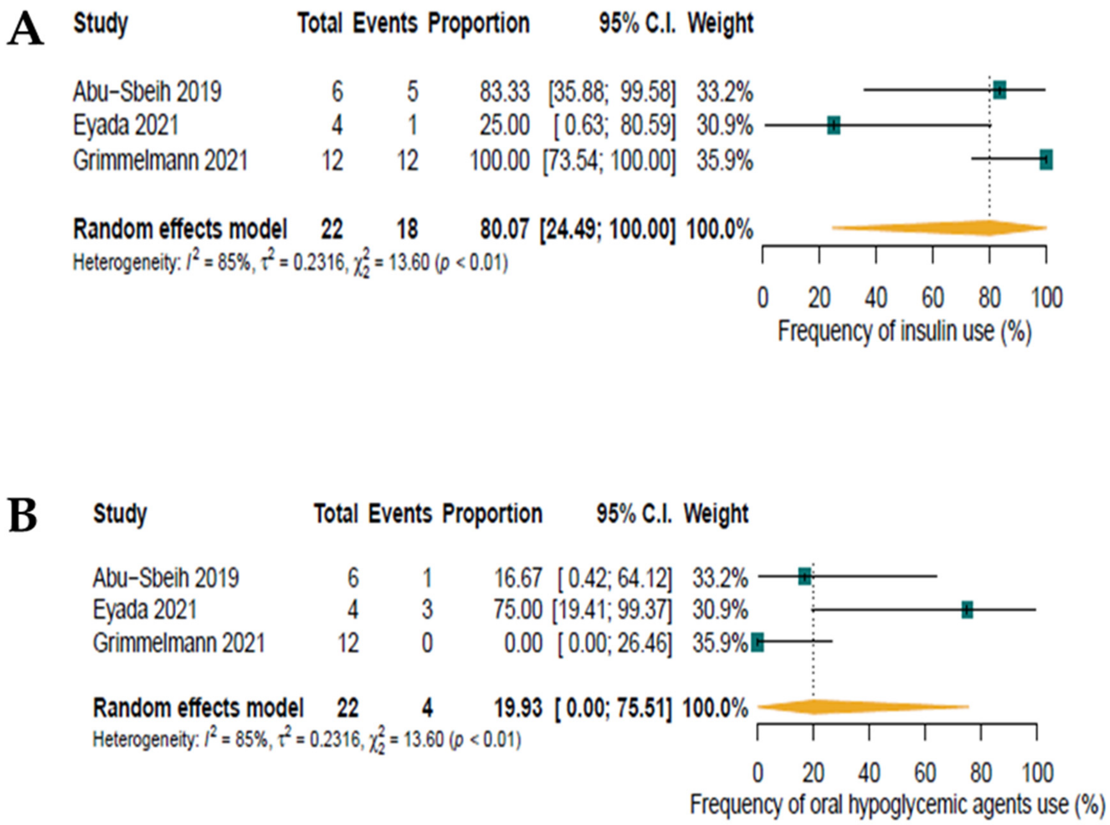

Figure S2: Management for long-term complication diabetes mellitus. A) insulin and B) oral hypoglycemic agents use.

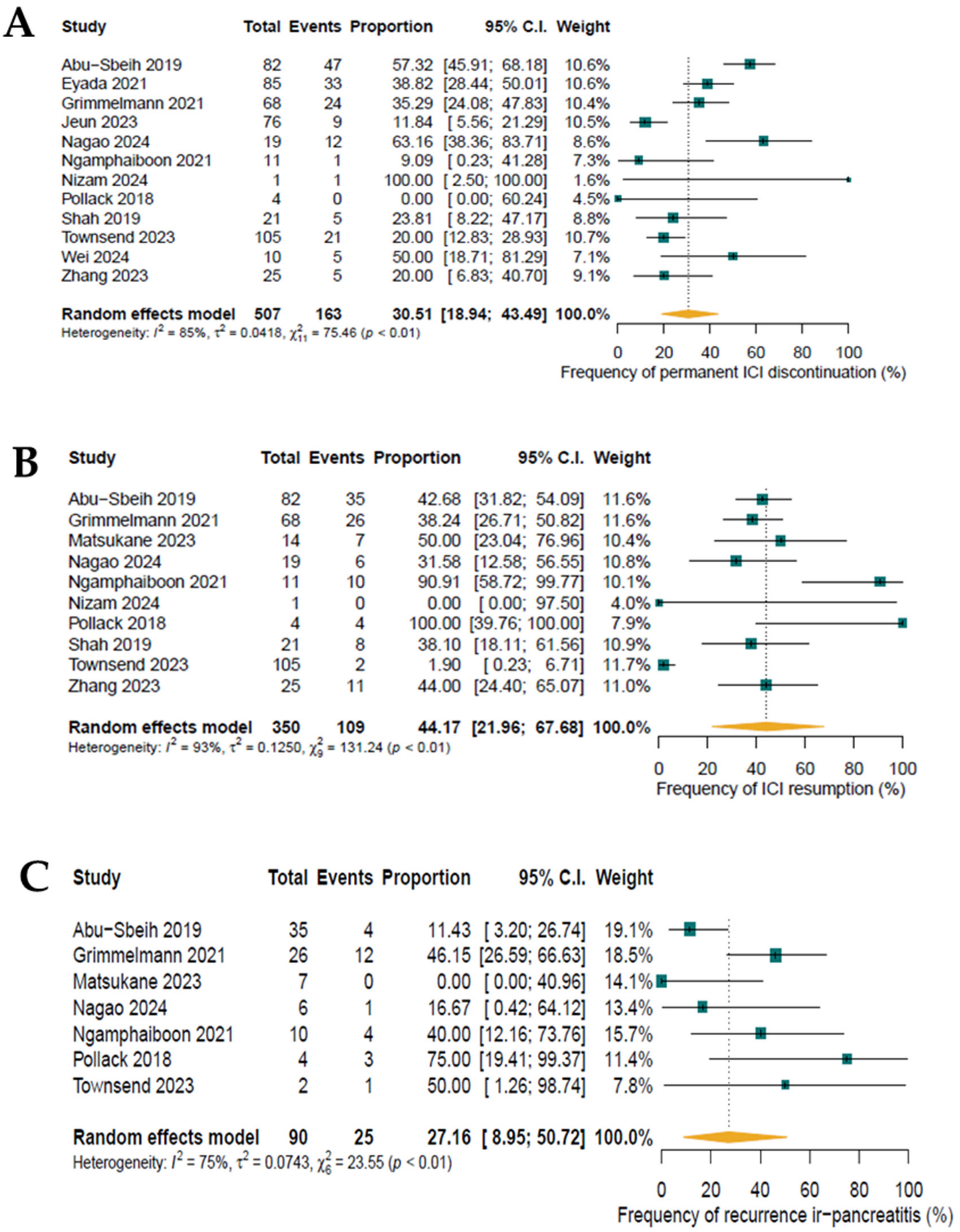

Figure S3: Frequency of (A) Permanent discontinuation of immune checkpoint inhibitors (ICI), (B) Resumption of ICI therapy, and (C) Recurrence of immune-related pancreatitis (ir-pancreatitis) following ICI therapy resumption.

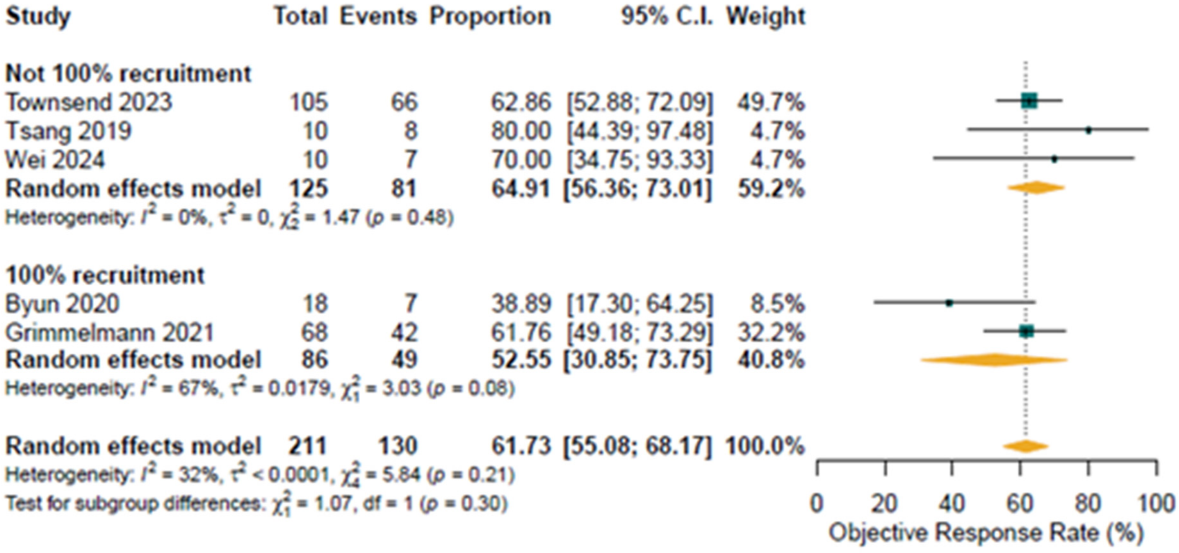

Figure S4: Objective response rate of patients with immune-related pancreatitis.

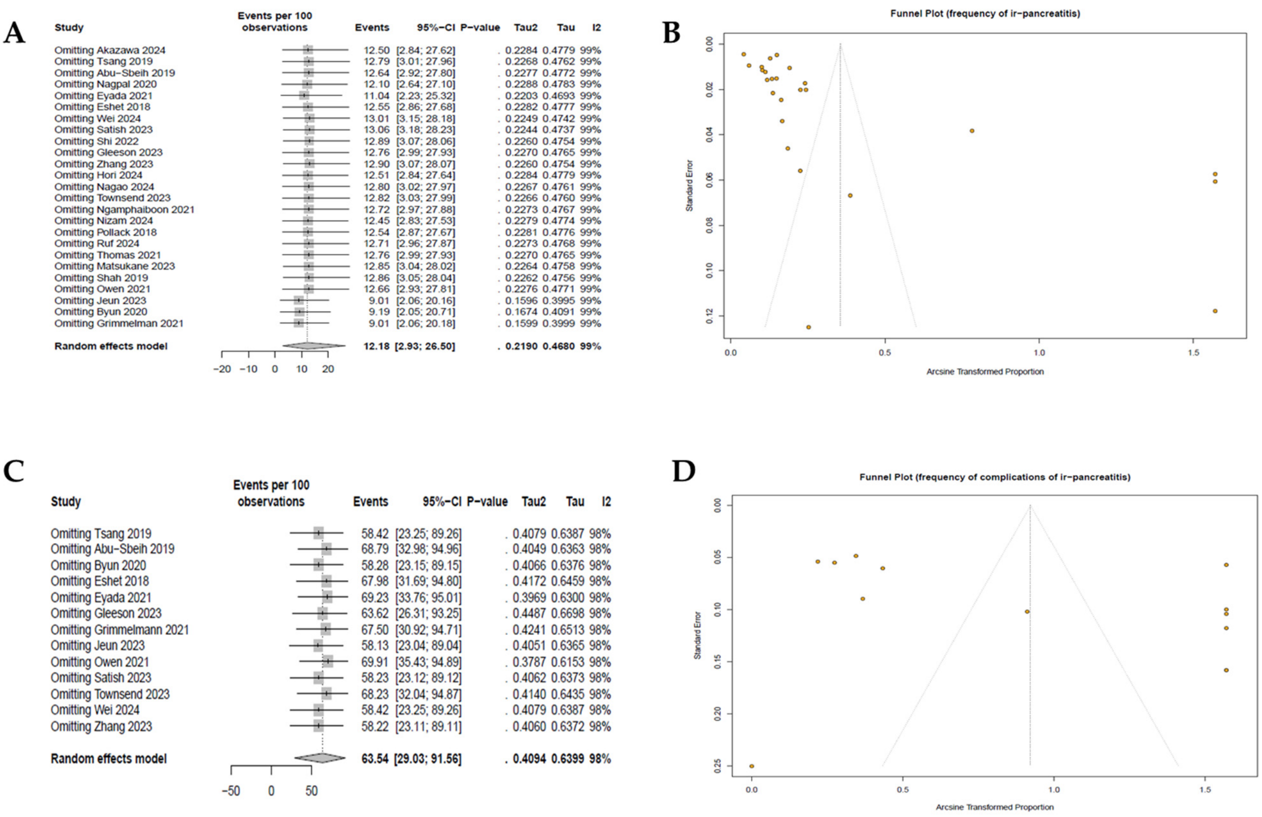

Figure S5: Leave-one-out and funnel plots of immune-related pancreatitis (ir-pancreatitis). (A & B) Frequency and (C & D) Chronic complications.

References

1. Abu-Sbeih, H.; Tang, T.; Lu, Y.; Thirumurthi, S.; Altan, M.; Jazaeri, A.A.; Dadu, R.; Coronel, E.; Wang, Y. Clinical Characteristics and Outcomes of Immune Checkpoint Inhibitor-Induced Pancreatic Injury. *j. immunotherapy cancer* **2019**, *7*, 31, doi:10.1186/s40425-019-0502-7.
2. Akazawa, Y.; Nosaka, T.; Takahashi, K.; Naito, T.; Matsuda, H.; Ohtani, M.; Nakamoto, Y. 967 ANALYSIS OF RISK FACTORS AND LONG-TERM PROGNOSIS IN PATIENTS WITH IMMUNE CHECKPOINT INHIBITOR-INDUCED PANCREATIC INJURY. *Gastroenterology* **2024**, *166*, S-231, doi:10.1016/S0016-5085(24)01016-3.
3. DJ, B.; R, B.; J, F.; J, Z.; RA, L.; S, K.; M, G. Immune Checkpoint Inhibitor-Associated Diabetes: A Single-Institution Experience. *Diabetes care* **2020**, *43*, 3106–3109, doi:10.2337/dc20-0609.
4. Eshet, Y.; Baruch, E.N.; Shapira-Frommer, R.; Steinberg-Silman, Y.; Kuznetsov, T.; Ben-Betzalel, G.; Daher, S.; Gluck, I.; Asher, N.; Apter, S.; et al. Clinical Significance of Pancreatic Atrophy Induced by Immune-Checkpoint Inhibitors: A Case–Control Study. *Cancer Immunol. Res.* **2018**, *6*, 1453–1458, doi:10.1158/2326-6066.CIR-17-0659.
5. Eyada, M.; Issac, A.; Abraham, F.; Jacob, J.S.; Chari, S.; Wang, Y.; Thomas, A.S. CLINICAL PROFILES OF ASYMPTOMATIC IMMUNE CHECK POINT INHIBITOR INDUCED PANCREATITIS (ICI-PI), A TYPE 3 AUTOIMMUNE PANCREATITIS. *Gastroenterology* **2021**, *160*, S-664, doi:10.1016/S0016-5085(21)02297-6.
6. Gleeson, F.C.; Kottschade, L.; Dunleavy, K.A.; Carr, R.M.; Hartgers, M.; Levy, M.J.; Ma, W.W.; McWilliams, R.; Egan, A. INCIDENCE AND EFFECT DURATION OF IMMUNE CHECKPOINT INHIBITOR RELATED PANCREAS ADVERSE EVENTS. *Gastroenterology* **2023**, *164*, S-1049, doi:10.1016/S0016-5085(23)03438-8.
7. Grimmelmann, I.; Momma, M.; Zimmer, L.; Hassel, J.C.; Heinzerling, L.; Pföhler, C.; Loquai, C.; Ruini, C.; Utikal, J.; Thoms, K.-M.; et al. Lipase Elevation and Type 1 Diabetes Mellitus Related to Immune Checkpoint Inhibitor Therapy – A Multicentre Study of 90 Patients from the German Dermatooncology Group. *European Journal of Cancer* **2021**, *149*, 1–10, doi:10.1016/j.ejca.2021.02.017.
8. Y, H.; I, N.; A, N.-I.; T, K.; M, Y.; A, K.; K, K.; H, S.; A, A.; T, T.; et al. Incidence of Pancreatic Injury and Pancreatitis in Patients Treated With Immune Checkpoint Inhibitors. *Clinical and translational gastroenterology* **2024**, *15*, e00667, doi:10.14309/ctg.0000000000000667.
9. R, J.; PC, I.; C, B.; V, L.; JM, V.; S, Y.; V, B.; IC, G.O.; R, D.; DR, M.; et al. Clinical Outcomes of Immune Checkpoint Inhibitor Diabetes Mellitus at a Comprehensive Cancer Center. *Immunotherapy* **2023**, *15*, 417–428, doi:10.2217/imt-2021-0316.
10. Matsukane, R.; Suetsugu, K.; Hata, K.; Matsuda, K.; Nakao, S.; Minami, H.; Watanabe, H.; Hirota, T.; Egashira, N.; Ieiri, I. Systematic Surveillance of Immune-Related Adverse Events in Clinical Practice and Impact of Subsequent Steroid Medication on Survival Outcomes. *Int. J. Clin. Oncol.* **2023**, *28*, 860–871, doi:10.1007/s10147-023-02349-3.
11. Nagao, K.; Sakai, A.; Tsumura, H.; Iemoto, T.; Hirata, Y.; Hori, H.; Ogisu, K.; Kakuyama, S.; Ikegawa, T.; Hirata, T.; et al. Pancreatic Injury in Patients Treated with Immune Checkpoint Inhibitors: A Retrospective Multicenterstudy. *J Gastroenterol* **2024**, *59*, 424–433, doi:10.1007/s00535-024-02083-1.
12. Nagpal, S.; Peeraphatdit, T.; Reid, P. CLINICAL CHARACTERISTICS OF PATIENTS WITH ATEZOLIZUMAB INDUCED PANCREATIC INJURY. *Gastroenterology* **2020**, *158*, S-872, doi:10.1016/S0016-5085(20)32865-1.
13. Ngamphaiboon, N.; Ithimakin, S.; Siripoon, T.; Sintawichai, N.; Sriuranpong, V. Patterns and Outcomes of Immune-Related Adverse Events in Solid Tumor Patients Treated with

Immune Checkpoint Inhibitors in Thailand: A Multicenter Analysis. *BMC Cancer* **2021**, *21*, doi:10.1186/s12885-021-09003-z.
14. Nizam, A.; Rader, R.K.; Tzeng, A.; Wei, W.; Sheng, I.Y.-F.; Martin, A.; Wee, C.E.; Gilligan, T.D.; Gupta, S.; Ornstein, M.C. Safety and Efficacy Outcomes in Immune Checkpoint Inhibitor-Treated Patients With Metastatic Urothelial Carcinoma Requiring Treatment Interruption or Discontinuation Due to Immune-Related Adverse Events. *Clin. Genitourin. Cancer* **2024**, *22*, 368–379, doi:10.1016/j.clgc.2023.12.007.
15. Owen, C.N.; Bai, X.; Quah, T.; Lo, S.N.; Allayous, C.; Callaghan, S.; Martínez-Vila, C.; Wallace, R.; Bhave, P.; Reijers, I.L.M.; et al. Delayed Immune-Related Adverse Events with Anti-PD-1-Based Immunotherapy in Melanoma. *Ann. Oncol.* **2021**, *32*, 917–925, doi:10.1016/j.annonc.2021.03.204.
16. Pollack, M.H.; Betof, A.; Dearden, H.; Rapazzo, K.; Valentine, I.; Brohl, A.S.; Ancell, K.K.; Long, G.V.; Menzies, A.M.; Eroglu, Z.; et al. Safety of Resuming Anti-PD-1 in Patients with Immune-Related Adverse Events (irAEs) during Combined Anti-CTLA-4 and Anti-PD1 in Metastatic Melanoma. *Ann. Oncol.* **2018**, *29*, 250–255, doi:10.1093/annonc/mdx642.
17. Ruf, T.; Kramer, R.; Forschner, A.; Leiter, U.; Meier, F.; Reinhardt, L.; Dücker, P.; Ertl, C.; Tomsitz, D.; Tietze, J.K.; et al. Second-Line Therapies for Steroid-Refractory Immune-Related Adverse Events in Patients Treated with Immune Checkpoint Inhibitors. *Eur. J. Cancer* **2024**, *203*, doi:10.1016/j.ejca.2024.114028.
18. Satish, D.; Lin, I.-H.; Flory, J.; Gerdes, H.; Postow, M.A.; Faleck, D.M. Exocrine Pancreatic Insufficiency Induced by Immune Checkpoint Inhibitors. *Oncologist* **2023**, *28*, 1085–1093, doi:10.1093/oncolo/oyad150.
19. Shah, R.; Sleiman, J.; Simons-Linares, R.; Faisal, M.; Song, J.-M.; Philpott, J.; Funchain, P. Variations of Diagnosis and Management of Immune Checkpoint Inhibitor Pancreatic Injury (ICIPI) and Immune Checkpoint Inhibitor Pancreatitis: A Single Institution Experience. *Am. J. Gastroenterol.* **2019**, *114*, S58, doi:10.14309/01.ajg.0000589904.13719.5f.
20. Shi, Y.; Fang, J.; Zhou, C.; Liu, A.; Wang, Y.; Meng, Q.; Ding, C.; Ai, B.; Gu, Y.; Yao, Y.; et al. Immune Checkpoint Inhibitor-Related Adverse Events in Lung Cancer: Real-World Incidence and Management Practices of 1905 Patients in China. *Thorac. Cancer* **2022**, *13*, 412–422, doi:10.1111/1759-7714.14274.
21. Shirwaikar Thomas, A.; Yedururi, S.; Eyada, M.; Wang, Y.; Chari, S.T. Symptomatic Type 3 Autoimmune Pancreatitis/Immune Checkpoint Inhibitor Pancreas Injury (ICI-PI) Leads to Chronic Pancreatic Injury. *Pancreas* **2021**, *50*, 1098, doi:10.1097/MPA.0000000000001904.
22. MJ, T.; M, L.; A, G.-H.; JS, S.; NR, L.; FS, H.; J, M.-B.; S, G. Pancreatitis and Hyperlipasemia in the Setting of Immune Checkpoint Inhibitor Therapy. *Journal of the National Comprehensive Cancer Network : JNCCN* **2023**, *21*, 831-840.e3, doi:10.6004/jnccn.2023.7034.
23. Tsang, V.H.M.; McGrath, R.T.; Clifton-Bligh, R.J.; Scolyer, R.A.; Jakrot, V.; Guminski, A.D.; Long, G.V.; Menzies, A.M. Checkpoint Inhibitor-Associated Autoimmune Diabetes Is Distinct from Type 1 Diabetes. *J. Clin. Endocrinol. Metab.* **2019**, *104*, 5499–5506, doi:10.1210/jc.2019-00423.
24. HH, W.; YC, L.; G, L.; CW, L.; YC, C.; JW, C.; MJ, L.; IW, C. Distinct Changes to Pancreatic Volume Rather than Pancreatic Autoantibody Positivity: Insights into Immune Checkpoint Inhibitors Induced Diabetes Mellitus. *Diabetology & metabolic syndrome* **2024**, *16*, 26, doi:10.1186/s13098-024-01263-6.
25. Zhang, Z.; Sharma, R.; Hamad, L.; Riebandt, G.; Attwood, K. Incidence of Diabetes Mellitus in Patients Treated with Immune Checkpoint Inhibitors (ICI) Therapy – A Comprehensive Cancer Center Experience. *Diabetes Res. Clin. Pract.* **2023**, *202*, doi:10.1016/j.diabres.2023.110776.
